# Evidence for Arousal-Biased Competition in Perceptual Learning

**DOI:** 10.3389/fpsyg.2012.00241

**Published:** 2012-07-19

**Authors:** Tae-Ho Lee, Laurent Itti, Mara Mather

**Affiliations:** ^1^Department of Psychology, University of Southern CaliforniaLos Angeles, CA, USA; ^2^Department of Computer Science, University of Southern CaliforniaLos Angeles, CA, USA; ^3^Davis School of Gerontology, University of Southern CaliforniaLos Angeles, CA, USA

**Keywords:** bottom-up salience, emotional arousal, optimal gain bias, pop-out search, threat, visual search

## Abstract

Arousal-biased competition theory predicts that arousal biases competition in favor of perceptually salient stimuli and against non-salient stimuli (Mather and Sutherland, 2011). The current study tested this hypothesis by having observers complete many trials in a visual search task in which the target either always was salient (a 55° tilted line among 80° distractors) or non-salient (a 55° tilted line among 50° distractors). Each participant completed one session in an emotional condition, in which visual search trials were preceded by negative arousing images, and one session in a non-emotional condition, in which the arousing images were replaced with neutral images (with session order counterbalanced). Test trials in which the target line had to be selected from among a set of lines with different tilts revealed that the emotional condition enhanced identification of the salient target line tilt but impaired identification of the non-salient target line tilt. Thus, arousal enhanced perceptual learning of salient stimuli but impaired perceptual learning of non-salient stimuli.

## Evidence for Arousal-Biased Competition in Perceptual Learning

Years of research have documented that emotion affects higher order cognitive processes such as decision making and memory in many ways (Bechara, 2004; Mather, 2007; Kensinger, 2009; Levine and Edelstein, 2009; Pessoa, 2009). More recent evidence indicates that emotion’s influence extends to perceptual processes as well, in part due to interactions between the amygdala and sensory cortices (for more details, see Phelps, 2006). For instance, Phelps et al. (2006) showed that presenting a fearful face rather than a neutral face could make a subsequent neutral stimulus (a Gabor patch) more easily perceived even at low contrast levels. Furthermore, Padmala and Pessoa (2008) showed that arousal-induced perceptual enhancements are associated with increased brain activation in area V1–V4 of the visual cortex. Also, seeing fearful faces can speed up people’s subsequent visual search (Becker, 2009; Olatunji et al., 2011; but see Quinlan and Johnson, 2011). However, emotion does not always enhance perceptual processing. For example, inserting an arousing distractor in a rapid serial visual presentation paradigm (RSVP) impairs identification of a subsequent neutral target stimulus (Most et al., 2005, 2006; Ciesielski et al., 2010).

**Figure 1:**
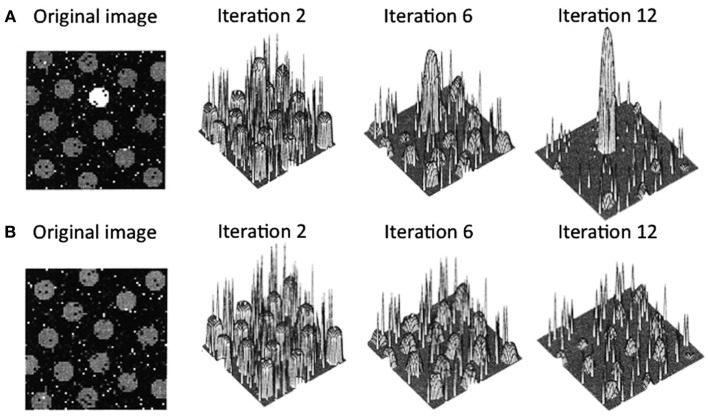
**Output from Itti and Koch’s (2000) computational saliency map model**. In case **(A)**, the original image has one location that is strongly activated by its bottom-up perceptual contrast surrounded by several locations with weaker initial activations. In this case, after an initial few iterations, the initial maximally activated location gains strength while suppressing the weaker activation locations. In case **(B)**, the original image has multiple locations that initially have similar activation levels. Here, all the peaks mutually suppress each other, leading to overall suppression of activation in the saliency map. Figure adapted from Itti and Koch (2000).

Arousal-biased competition theory attempts to explain how arousal can both enhance and impair perception and memory (Mather and Sutherland, 2011; Sutherland and Mather, 2012). The theory builds on models of biased competition (Bundesen, 1990; Desimone and Duncan, 1995; Miller and Cohen, 2001; Deco and Rolls, 2005; Beck and Kastner, 2009) by positing that arousal amplifies biased competition processes, leading to “winner-take-more” and “loser-take-less” effects. More specifically, arousal-biased competition theory builds on a computational model of visual attention (Itti and Koch, 2000), which proposes that incoming information is first analyzed by early visual neurons to represent the perceptual contrast at each location for a variety of elementary visual features (e.g., luminance, color, orientation, motion, etc.). Within each of these feature maps, locations compete for activation via a center-surround competitive process in which excitation at a particular location leads to further excitation at that location while suppressing its surrounding locations. As depicted in Figure 1A, if one location starts with higher activity than the other locations, after several iterations, that location will dominate the map even more. In contrast (Figure 1B), if several locations in the map have similar initial activation levels due to similar perceptual contrast, these regions will be mutually suppressed. The contrast values across individual feature maps (e.g., individual maps for luminance, color, etc.) are integrated to obtain the overall saliency at each location.

According to arousal-biased competition theory, arousal increases the impact of these competitive processes, such that when there is one salient location (e.g., Figure 1A), that location will gain even more activation than under non-arousing conditions. This should lead to enhanced processing of the stimulus in that location, increasing learning about it and increasing the specificity of neural representations of that stimulus. In contrast, in situations with multiple similar competitors (e.g., Figure 1B), arousal will lead to even greater suppression of all initially active locations, impairing processing of any stimulus in one of those locations and decreasing the specificity of neural representations of that stimulus compared with non-arousing situations.

**Figure 2:**
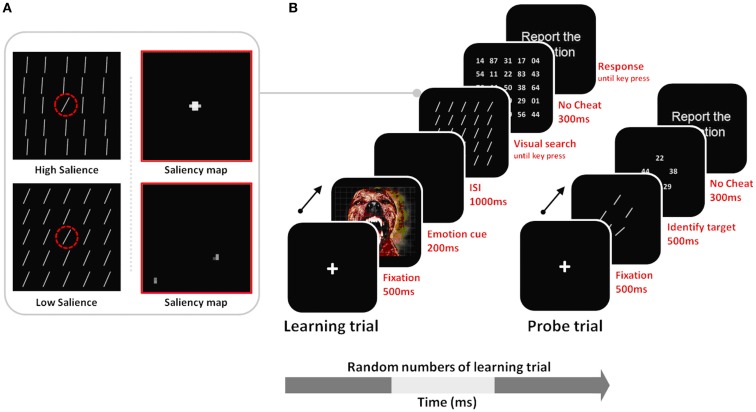
**(A)** Stimulus examples of low vs. high salience targets with their corresponding saliency maps derived from Itti and Koch’s (2000) computational model. For the high-salience condition, the distractor lines were tilted at 80°, creating a 25° difference between them and the 55° target; therefore iterative spatial competition leads the target’s location to gain further strength while suppressing surrounding regions. In the low-salience condition, the target and the distractor differ in tilt only by 5°, and therefore the similarly activated locations in the saliency map inhibit each other, leading to mutual suppression of all locations in the saliency map. The dashed red circle indicates the target location, but it was not seen by participants. **(B)** Learning trials involved visual search for the 55° target, allowing perceptual learning about that target; probe trials were interspersed with the learning trials and tested recognition of the target tilt as observers had to select the 55° target from among a set of five differently tilted lines. Note that stimuli are not drawn to scale here.

In the current study, we tested these hypotheses in the domain of visual search, examining how arousal affects perceptual learning of salient targets versus non-salient targets. We adapted the general outline of a procedure in a previous study (Navalpakkam and Itti, 2007). In our version of the search task, we included both low and high-salience target conditions, and both arousing and non-arousing sessions (Figure 2). During the learning trials of the task, participants were trained to detect a target line oriented at 55° among 24 distractors oriented either at 50° (in the low-salience condition) or at 80° (in the high-salience condition). To test learning of the tilt of the target line, probe trials were interspersed in a random manner between learning trials. The probe trials had five different lines in a circular array, and, as in Navalpakkam and Itti (2007), participants’ task was to find the target line. To investigate the effects of arousal on learning performance, negative arousing, or neutral non-arousing pictures were presented before stimuli arrays in the learning phase.

In early visual cortex, neurons represent specific sensory features (Hubel and Wiesel, 1959). For instance, one neuron might fire most in response to a line tilted at 55° whereas another neuron might fire most in response to an 80° line (Somers et al., 1995). Neural selectivity is imperfect, in part due to noise, such that a neuron stimulated most by 55° tilted lines still fires – at a less intense rate – to similarly tilted lines. The plot of a neuron’s average firing rate as a function of stimulus parameters such as tilt orientation is known as its “tuning curve” (Solla, 2006). Neuronal tuning curves shift as a result of experience and learning (e.g., Yang and Maunsell, 2004). While behavioral responses cannot indicate the specific responses at the neuronal level, they can provide analogous psychophysical tuning curves that reveal the accuracy and specificity of the neural representation of a particular feature or stimulus (e.g., Lee et al., 1999). In the current study, we used the data from the probe trials to assess the target line memory representations and to model the tuning curves associated with these representations.

We predicted that we would observe arousal effects on perceptual representations as a function of target prominence and the competitive processes enhancing high-salience stimuli and suppressing low-salience stimuli. Specifically, in the high-salience condition, we predicted that experiencing arousal would enhance perceptual learning of the highly salient target features. As competitive processes between stimuli representations influence the variability or noise in the perceptual representations as well as their signal strength (Ling and Blake, 2009), we predicted that the enhanced perceptual learning would be evident in decreased noise in the tuning curves (evident in decreased bandwidth of the curves) as well as in increased amplitude of the tuning curves at the correct 55° point. In contrast, in the low-salience condition, we predicted that arousal would impair learning target features, decreasing amplitude, and increasing noise.

In addition, in the low-salience condition, Navalpakkam and Itti 2007) documented an interesting phenomenon they called “optimal feature gain,” in which the neural tuning curve that represents the target is shifted away from the distractor features, when the target and distractors are similar. Thus, for instance, when the participants completed visual search trials in which the target was a 55° line seen among 50° distractors, Navalpakkam and Itti found that the peak amplitude of participants’ tuning curves for the target was not 55°, as might be expected, but instead was shifted to 60°. This shift in representation away from the distractor optimizes discrimination because the 55° target and 50° distractor now fell on a region of the tuning curve that has a higher slope than the peak of the curve, and similar stimuli are most easily discriminated in high-slope regions of a tuning curve. However, this discrimination advantage for high-slope regions of tuning curves disappears with increasing noise level in the representation (Butts and Goldman, 2006). Thus, given that we predicted that arousal would make it harder to distinguish the non-salient target from its distractors because of increased noise in the tuning curve for the target, arousal should also reduce the likelihood that participants will show ”optimal feature gain” in the low-salience condition.

## Materials and Methods

### Observers and psychophysical sessions

Twenty observers (10 males, 10 females; ages 25–36) with corrected-to-normal vision volunteered for this study and gave informed consent. Observers were naïve to the purpose of the experiment (except one, TL).

Ten (six males and four females) were assigned to the high-salience and the other ten (four males and six females) to the low-salience condition. For each salience type, observers completed two emotion sessions (arousing and non-arousing) in a counterbalanced order.

### Stimuli and apparatus

Line stimuli consisted of five types of line orientation (30°, 50°, 55°, 60°, and 80°). The images used in the learning trials (32 negative images for the arousing session and 32 neutral images for the non-arousing session) and the additional images used in the subsequent memory task (32 negative and 32 neutral) were selected from the International Affective Pictures System (IAPS: Lang et al., 1999) and the Mather and Nesmith stimulus set (Mather and Nesmith, 2008). Nine additional participants rated the images for arousal (on a scale of 1 = calm to 9 = arousing) and valence (on a scale of 1 = unpleasant to 9 = pleasant). The 32 negative images had more negative valence (*M* = 1.97, SE = 0.38) and higher arousal ratings (*M* = 7.77, SE = 0.41) than the 32 non-arousing images (*M* valence = 5.45, SE = 0.33; *M* arousal = 1.88, SE = 0.38). The size of each line stimulus and emotional images corresponded to 1.5° × 0.6° and 30.5° × 22.5° visual angles, respectively. The stimuli were displayed on a 19′′ CRT monitor with a refresh rate of 100 Hz. All observers were tested individually in a soundproof room, seated approximately 65 cm away from a screen, using a chin-rest.

### Procedure

As shown in Figure 2A, observers performed both learning trials and probe trials. Every so often, after a random number of learning trials, knowledge about the target was measured in a probe trial. Learning trials proceeded as follows: (A) A 500-ms fixation cross display; (B) a 200 ms-emotional picture; (C) a 1000 ms blank screen; (D) a search array containing one target (55°) among 24 distractors. Based on the salience type assigned to observers, the target line was presented among distractors tilted either 80° or 50° (see Figure 2A). To manipulate observers’ arousal levels during the session, we presented pictures in an approximately 60% partial schedule in both the arousing and non-arousing sessions. In trials without a picture, the search display was presented right after the first 500-ms fixation event.

Observers were instructed to find the target (55°) and press any key. To verify that observers indeed found the target on every trial, following the key press, a grid of fine-print numbers appeared briefly (300 ms) and observers were asked to report the number at the target’s location (Figure 2B). Feedback (“correct” or “incorrect”) on performance was given after each trial. After a random number of learning trials, a probe trial was presented. The probe trial consisted of a 500-ms fixation display, followed by a 500-ms display of five items representing five lines (30°, 50°, 55°, 60°, and 80°) within a 6.0° × 6.0° rectangular box, and then by a 300-ms display of five fine-print random numbers. The task was the same as in the learning trials. Observers were asked to report the number at the target location. Observers first completed 14 trials in a practice session, followed by the main task phase. Both sessions started with these practice trials and in both cases, no emotional pictures were shown during the practice session.

The line-search task consisted of ten 50-trial blocks (each with 34 learning trials and 16 probe trials). Each observer performed the task with 160 probe trials randomly presented in between 340 learning trials for each session (arousing and non-arousing). Thus, 1000 trials (2 emotion sessions × 50 trials × 10 blocks) were administered for each observer. Each observer either saw all low-salience or all high-salience targets. Observers were allowed to take a break in between blocks. The order of emotion sessions was randomly assigned across the observers. To avoid learning effects across sessions for the target line, we adopted two different orientations (original and reversed). For example, when the observer performed and completed the first session with the original orientation (e.g., 55°), the second session was administered with the reversed orientation (e.g., 125°). Immediately after each session, observers performed a recognition memory task as a manipulation check that they processed the pictures. For the recognition task, a randomly selected half of the main task images served as old items intermixed with 16 new images. The old and new items were presented in a random order and the observer was asked to indicate “old” or “new” for each image.

## Results

### Probe trial performance

**Figure 3:**
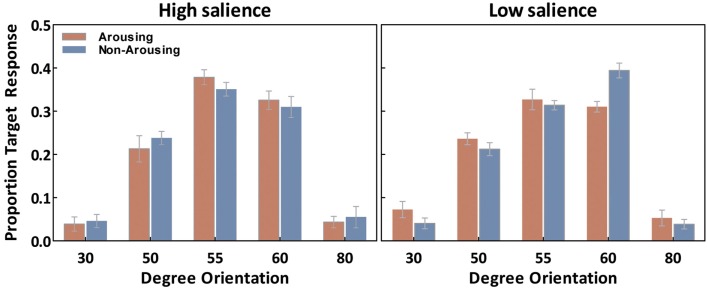
**Averaged “target” responses for each orientation in the probe trials as a function of emotion and salience**. Error bars represent SEM.

We first examine our measure of interest, the ability to correctly recognize the exact tilt of the target line in each of the conditions. For each observer, the percentage of “target” responses on probe trials was calculated for each orientation (30°, 50°, 55°, 60°, and 80°) separately for each emotion session (Figure 3). These were analyzed with salience type (2: high- and low-salience) as between-subject variables, and session (2: arousing and non-arousing) and orientation (5: 30°, 50°, 55°, 60°, and 80°) as within-subject variables. There was a significant main effect of orientation, *F*(4,72) = 145.20, *p* < 0.001, η^2^ = 0.89, and a salience × emotion × orientation interaction, *F*(4,72) = 3.90, *p* < 0.01, η^2^ = 0.18. Subsequent simple-effects analyses comparing performance in the two session types revealed that, in the high-salience condition, participants selected the correct target (i.e., 55° responses) more frequently in the arousing condition than in the non-arousing condition (*p* < 0.05). In contrast, in the low-salience condition, emotion condition did not significantly affect the percent of responses identifying the correct target. However, the arousing condition led to a significant decrease in selecting the 60° target (or its corresponding opposite line in the flipped condition; *p* < 0.001) in the low-salience condition.

To understand the nature of these results better, we estimated each observer’s tuning curve to fit responses from each emotion session via a Gaussian function known to be well represented in tuning curves:

(1) $$f\left(x\right)=ae^{\frac{-{\left(x-\mu \right)}^{2}}{2\sigma^{2}}}$$

where *a* represents response amplitude (i.e., the height of the curve’s peak), μ specifies the position of the center of the peak, and σ is the bandwidth (i.e., standard deviation of the curve). The goodness of fit was evaluated by the *r*^2^ for each arousing condition and non-arousing condition:

(2) $$r^{2}=1.0-\frac{\sum {\left(y_{i}^{\mathrm{Pr}\text{edicted}}-y_{i}^{\text{Observed}}\right)}^{2}}{{\sum \left[y_{i}^{\text{Observed}}-\text{mean(}y_{i}^{\text{Observed}}\text{)}\right]}^{2}}$$

To evaluate the curve fit model using the parametric values (i.e., *a*, μ, and σ) for each condition, a nested model testing (separate fits for each emotion condition vs. one fit for both conditions collapsed together) was applied. Specifically, an *F*-test for nested models was used to statistically compare the models based on the averaged *r*^2^s for the arousing and non-arousing conditions. For two nested models with *k*_full_ and *k*_reduced_ parameters, the *F* statistic is defined as:

(3) $$F\left(df_{1},df_{2}\right)=\frac{\left(r_{\text{full}}^{2}-r_{\text{reduced}}^{2}\right)/df_{1}}{\left(1-r_{\text{full}}^{2}\right)/df_{2}}$$

where *df*_1_ = *k*_full_ − *k*_reduced_, and *df*_2_ = *N* − *k*_full_; *N* is the number of data points. All these procedures were performed using the GraphPad Prism version 5.04 for Windows (GraphPad Prism Software, La Jolla, CA, USA; see also Motulsky and Christopoulos, 2004).

**Figure 4:**
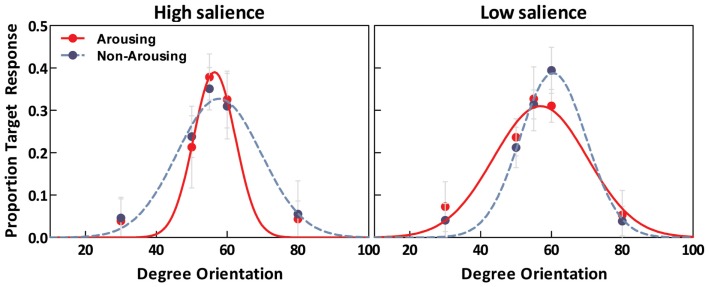
**Estimated tuning curves for averaged “target” responses as a function of emotion in the high-salience condition (left) and low-salience condition (right)**.

As illustrated in Figure 4, estimated tuning curves for the averaged “target” responses across all observers revealed that emotional arousal modulated response patterns differently depending on salience. When the target was conspicuous among distractors (i.e., high-salience condition), arousal enhanced the accuracy and strength of the target’s representation; this was evident in the decreased bandwidth, *F*(1,94) = 4.91, *p* < 0.05, and increased amplitude, *F*(1,94) = 4.71, *p* < 0.05. On the contrary, when target salience was low, arousal widened the tuning curve leading to specificity loss. This was evidenced by increased bandwidth, *F*(1,94) = 8.86, *p* < 0.005, and decreased amplitude, *F*(1,94) = 13.85, *p* < 0.0005. The position of the peak amplitude also shifted, *F*(1,94) = 7.03, *p* < 0.01. This shift in the position of the peak amplitude indicated that when target salience was low, arousal also disrupted the “optimal feature gain” exaggeration of target-distractor differences seen in the non-arousing condition and in a previous study not involving emotion (Navalpakkam and Itti, 2007). The parameters of the best fitting functions are listed in Table 1,^1^. In the following sections, we describe performance on the other aspects of the task.

**Table 1:** **Parameters of the best fit for the averaged “target” response in probe trials for the arousing versus the non-arousing sessions and *p* values from the comparisons of each parameter using nested model testing**.

Saliency	Parameters	Emotion	
		Arousing	None
High	μ	56.46	57.77
	*a*	0.39	0.33*
	σ	5.89	11.87*
Low	μ	56.94	60.51***
	*a*	0.31	0.39****
	σ	13.27	9.35***

****p < 0.0005, ***p < 0.005, **p < 0.01, *p < 0.05.

### Memory for the pictures

An analysis of variance (ANOVA) with salience type (2: high- vs. low-salience) as a between-observers variable and session type (2: arousing vs. non-arousing) as a within-observers variable revealed that observers’ *d*-prime (*d*′) values from the picture recognition memory task were significantly higher in the arousing picture sessions (*d*′ = 3.37, SE = 0.09) than in the non-arousing picture sessions (*d*′ = 2.80, SE = 0.14), *F*(1,18) = 8.58, *p* < 0.001, η^2^ = 0.52. There was no significant main effect of salience type nor interaction with salience type (both *p* > 0.3). Thus, as seen across many previous studies, memory was better for the emotional pictures than the neutral pictures (for a review see Reisberg and Hertel, 2004). For the purposes of the current study, however, the relevant finding was that participants had similar memory for the pictures across the two salience conditions.

### Learning trial performance

**Figure 5:**
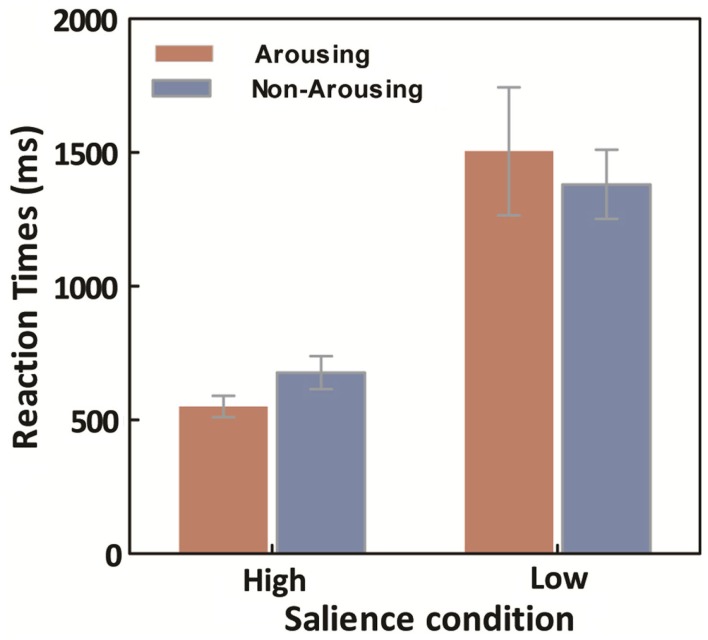
**Average across participants of within-participant median learning-trial response times, as a function of emotion and salience**. Error bars represent SEM.

Averaged median response times (RTs) for the learning trials were calculated for each session for both high- and low-salience conditions. A repeated ANOVA on target search latencies was conducted with salience type (2: high- vs. low-salience) as a between-observers variable, and session type (2: arousing vs. non-arousing) as a within-observers variable. Not surprisingly, there was a main effect of salience type, *F*(1,18) = 134.17, *p* < 0.001, η^2^ = 0.88, with slower RTs in the low-salience condition (*M* = 1443.45, SE = 132.16) than in the high-salience condition (*M* = 581.53, SE = 36.37). However, there was no significant main effect of session type and no significant interaction between the two variables (Figure 5).

**Figure 6:**
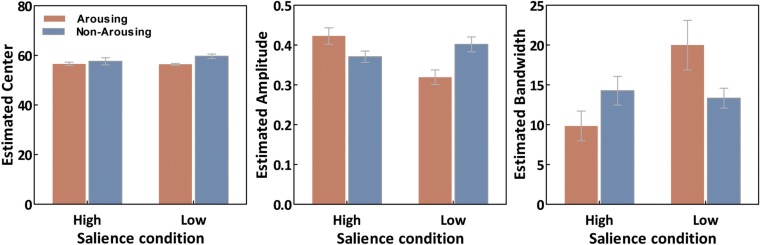
**Averaged curve position (μ), amplitude (*a*), and bandwidth (σ) of tuning curves as a function of conditions**. Error bars represent SEM.

Overall, observers had near-ceiling accuracy (*M* = 0.977, SE = 0.005) on the learning trials. More specifically, in the high-salience condition, the averaged correct ratio was 0.991 in arousing condition and 0.988 in non-arousing condition. In the low-salience condition, the mean was 0.975 in the arousing condition, and 0.956 in the non-arousing condition. A repeated-measures ANOVA with salience type (2: high- vs. low-salience) as a between-observers variable and session type (2: arousing vs. non-arousing) as a within-observers variable revealed that there was a main effect of session type, *F*(1,18) = 11.12, *p* < 0.005, η^2^ = 0.38, and a main effect of salience type, *F*(1,18) = 10.27, *p* < 0.005, η^2^ = 0.36. There was an interaction with salience type, *F*(1,18) = 5.63, *p* < 0.05, η^2^ = 0.24. Subsequent simple-effects analyses for each salience type across the two session types revealed that, in the low-salience condition, the correct ratio was significantly greater in the arousing condition than in the non-arousing condition (*p* < 0.05). In contrast, in the high-salience condition, emotion condition did not significantly affect the percent of responses identifying the correct target (*p* > 0.1). However, it is not clear if this interaction is simply an artifact of the near-ceiling accuracy in the high-salience condition, as the near-perfect accuracy in this condition may have diminished the effects of arousal on accuracy (which appear to be in the direction of enhancing performance, as in the low-salience condition). In summary, arousal generally increased accuracy in the search task, even in the low-salience condition in which arousal impaired perceptual learning about the target.

### Comparing learning trials preceded by pictures to those not preceded by pictures

In our study, although emotion type was manipulated across sessions, within each session we did not show a picture on every trial. To provide more information about whether the presence or absence of an image on a particular learning trial mattered for the speed of the response, we conducted a follow-up ANOVA with salience type (2: high- vs. low-salience) as between-observer variables, and image presence (2: image present before visual search, vs. image absent) and session (2: arousing vs. non-arousing) as within-observer variables, and the learning phase median RT as the dependent variable. There was a significant interaction of image presence (2: presence vs. absent) and salience condition (2: high vs. low-salience), *F*(1, 18) = 8.00, *p* < 0.05, η^2^ = 0.31. However, there was no session main effect, *F*(1, 18) = 0.84, n.s., nor any interactions with session (*P*s > 0.4). To clarify the nature of the image presence and salience condition interaction, we carried out a separate repeated ANOVA for each salience condition with image presence as a factor. There were no statistically significant effects in the high-salience condition (*P*s > 0.1). In contrast, in the low-salience condition, there was a main effect of image presence, *F*(1,9) = 11.06, *p* < 0.01, η^2^ = 0.55. In this condition, the RT was generally slower with an image absent (*M* = 1592.90 ms, SE = 199.05) than present (*M* = 1345 ms, SE = 147.49) regardless of emotion condition. However, there was no interaction or main effect of session. Thus, in addition to not detecting session differences in reaction times during the learning phase, we did not detect trial-by-trial differences in reaction time based on whether the picture was emotional or not – indicating emotion did not significantly influence response speed in the learning trials.

## Discussion

In this study, we tested the hypothesis that arousing stimuli increase the effects of competition among stimuli in perceptual learning. We compared the effects of arousal in two types of visual search situations. In the high-salience condition, the target line was tilted 55° and the distractor lines were tilted 80°. In this type of visual display, the target had high perceptual contrast with the surrounding stimuli and so center-surround competition should increase the perceptual salience of the target compared with its surrounding stimuli (Itti and Koch, 2000). Arousal-biased competition theory (Mather and Sutherland, 2011) predicts that arousal should further increase the activation of this perceptual “winner,” making it more precisely represented and encoded.

In the high-salience condition, when asked to identify which of five alternative lines was the target discrepant line in the visual search trials, in both the arousing and non-arousing sessions participants were most likely to select the correct 55° tilted line. However, in the arousing session, participants were significantly more likely to select the correct 55° option than the other options, leading to a higher amplitude and a lower bandwidth for their psychophysical tuning curve representing the target line tilt.

In the low-salience condition, the target line (tilted 55°) and the distractor lines (tilted 50°) were similar. In this situation of competition between stimuli with similar perceptual contrast, center-surround competition mechanisms should mutually inhibit both target and distractor locations (Itti and Koch, 2000). If, as predicted by arousal-biased competition, arousal amplifies the effects of these competition processes, then learning of low-salience targets should be worse under arousing than non-arousing situations. Consistent with these predictions, in the arousing sessions, observers learned the target line tilt less precisely than in the non-arousing sessions. Thus, emotional arousal had opposite effects on perceptual learning of salient and non-salient stimuli. Previous research indicates that competitive processes in binocular rivalry lead not only to relative differences in signal strength between the dominant and suppressed stimuli, but also to less noise in the representation of the dominant stimulus than in the representation of the suppressed stimulus (Ling and Blake, 2009). Consistent with this, in our study, arousal decreased the noise in the tuning curves of salient stimuli but increased it for non-salient stimuli.

Previous studies have shown that if people see emotionally arousing pictures while they are trying to remember several neutral stimuli, they are less able to recognize the neutral stimuli at the end of the trial (Dolcos and McCarthy, 2006; Dolcos et al., 2006). However, impaired working memory between learning and probe trials cannot account for our findings, as in the high-salience condition, arousal enhanced memory for the target line. Instead, arousal-biased competition provides a framework to account for when arousal will impair working memory and when it will enhance it. The prediction is that arousal will impair working memory when multiple equally salient stimuli are competing for representation, such as on working memory trials with several neutral faces as the memoranda and distracting arousing or neutral pictures (Dolcos and McCarthy, 2006; Dolcos et al., 2006). Arousal can even impair memory for associated features of arousing stimuli when the features of multiple arousing stimuli are competing for representation (Mather et al., 2006; Mitchell et al., 2006). However, when arousing stimuli compete with neutral stimuli in an N-back working memory task, the arousing stimuli, which presumably have higher priority due to both salience and goal-relevance, are remembered better than the neutral stimuli (Lindstrom and Bohlin, 2011).

Research on perception reveals similar issues regarding how arousing stimuli can both modulate competition among independent neutral stimuli and also compete directly against those stimuli. For instance, previous research indicates that arousing stimuli such as fearful faces can enhance perception of subsequent neutral stimuli (e.g., Phelps et al., 2006; Padmala and Pessoa, 2008). However, these studies did not evaluate how arousal affected the competition among more and less salient stimuli. The prediction from our study is that arousal would enhance perception only of the most salient stimuli while impairing perception of less salient stimuli. But a critical issue here, as in the working memory studies, is that arousing stimuli also compete for representation. Thus, when pictures are rapidly displayed in a sequence, arousing pictures impair perception of subsequent targets (Most et al., 2005, 2006, 2007; Smith et al., 2006; Most and Junge, 2008; Ciesielski et al., 2010; Wang et al., in press). The timing between a cue inducing arousal and a subsequent neutral target is critical in determining whether the arousing cue itself dominates everything else, or whether it can enhance perception of a salient target. For instance, in one study (Bocanegra and Zeelenberg, 2009), when the interval between the cue and the target was 50 or 500 ms, participants were less likely to correctly identify the target when the cue was arousing. However, increasing the interval to 1000 ms led to enhanced identification of targets following arousing cues. In our study, the intertrial interval was 1000 ms, at which point the arousing stimulus was no longer in direct competition with subsequent stimuli.

It is interesting that we did not see any effects of arousing stimuli on RTs to detect the visual search target, whereas two previous studies (Becker, 2009; Olatunji et al., 2011) found that showing fearful faces 600 ms or immediately before a search array enhanced target detection. Olatunji et al. found that this advantage was specific to fear face cues and did not appear for anger or disgust face cues. Thus, it may be that the enhanced search detection is specific to fear and so was not elicited by the mixed negative emotionally arousing pictures we showed. Furthermore, it is worth noting that enhanced visual search after fearful face cues was not replicated in another study (Quinlan and Johnson, 2011). In any case, the fact that we did not see significant effects of arousal on visual search speed rules out the possibility that the perceptual learning effects we found were mediated by target detection speed differences across emotion conditions. Also, search accuracy did not show arousal-biased competition effects; instead arousal seemed to have a general enhancing effect on initial search accuracy, which may have been due to enhancing effects of arousal on sustained attention. The lack of arousal-biased competition effects in initial search speed or accuracy suggests that the differences in perceptual learning induced by emotional arousal were due to competitive processes acting on representations after target detection.

In the current study, there was an additional interesting finding in the low-salience condition. Here, the visual search parameters were the same as in Navalpakkam and Itti’s (2007) study, in which they found evidence that, in difficult search without any emotion induction, people shift their perceptual representation of the target item such that it is less accurate, but more optimal for discriminating the target from its distractors. Standard models of attention assume that attention increases the activity of neurons tuned to respond to the target’s features (Carrasco, 2011). Navalpakkam and Itti modeled situations in which the target and the distractors are highly similar, such as search for a 55° target among 50° distractors. Their model suggests that boosting activity of neurons tuned for the exact target feature can be suboptimal when the target and distractors are very similar. In this case, the optimal strategy is to increase the signal strength of neurons representing features like the target, but that differ more from the distractors than the target does. In the case of a 55° target among 50° distractors, this would mean it would be optimal to boost the responsiveness of neurons tuned to respond to 60° lines, as these neurons should have the steepest part of their tuning curve coincide with the small differences in the feature value between the target and distractor (see Purushothaman and Bradley, 2005).

Navalpakkam and Itti confirmed their model in behavioral studies in which people showed this “optimal feature gain” strategy when learning the features of targets that were very similar to distractors. This strategy requires relatively sharp tuning curves, as with broader tuning curves there would be little difference in the tuning curve slope height at 50° (the distractor) between neurons tuned for 55° and 60° lines. Indeed, other modeling work indicates that similar stimuli are most easily discriminated in high-slope regions of the tuning curve only when there are low noise levels in tuning curves (Butts and Goldman, 2006). In our study, we replicated Navalpakkam and Itti’s “optimal feature gain” effects in the non-arousing low-salience condition, such that observers were more likely to incorrectly identify the target as having a 60° tilt rather than its actual 55° tilt. However, in the arousing condition, representations of the target line were significantly less shifted away from the distractor tilt, and revealed a significantly broader tuning curve with lower amplitude. This finding suggests that, in difficult discrimination tasks involving similar targets and distractors, emotional arousal disrupts people’s ability to make subtle shifts in perceptual representations that optimize discrimination of targets from distractors.

We used negative stimuli in our study as they generally induce stronger arousal responses than positive stimuli (Lang et al., 1998; Baumeister et al., 2001). However, this means that we cannot be sure whether our results are due to the effects of negative valence or emotional arousal. Previous research reveals that highly arousing positive and negative stimuli affect subsequent perceptual processing in similar ways; for instance, like negative arousing pictures, erotic pictures impair perception of visual targets (Most et al., 2007). However, additional research is needed to test whether, like negative arousing stimuli, positive arousing stimuli amplify biased competition processes.

One of the most critical aspects of our perceptual processes is that they allow us to be selective about what we attend to. Being able to focus on some aspects of incoming perceptual stimuli while ignoring others is critical for being able to process and respond to high priority stimuli in the environment. Perceptual contrast is one cue that helps determine priority. Stimuli that move suddenly or are brighter than their surroundings are salient and win out over other stimuli to draw attention. Our study suggests that when people experience negative emotional arousal, these competitive processes are amplified such that salient stimuli are represented even more and non-salient stimuli even less accurately than they would be otherwise. Such processes should enable the type of focused processing necessary under threatening or critical circumstances, but they come at the cost of reduced learning about non-salient information.

## Conflict of Interest Statement

The authors declare that the research was conducted in the absence of any commercial or financial relationships that could be construed as a potential conflict of interest.

## Footnotes

^1^The estimated parameters were compared via a repeated-measures ANOVA which revealed the same pattern of results as nested model testing (Figure 6). For the curve position parameter (i.e., μ), there was a main effect of emotion, *F*(1,18) = 9.05, *p* < 0.01, and an interaction between emotion and salience *F*(1,18) = 7.87, *p* < 0.05. For the curve amplitude parameter (i.e., *a*), there was a significant emotion by salience interaction, *F*(1,18) = 12.283, *p* < 0.005. The curve bandwidth parameter (i.e., σ) also showed a significant emotion by salience interaction, *F*(1,18) = 8.99, *p* < 0.01.
